# Virulence Profile: Renate König

**DOI:** 10.1080/21505594.2017.1388653

**Published:** 2017-11-24

**Authors:** Renate König

## When did you first get interested in science?

From an early age already, my parents had instilled in me the love for nature and wildlife. On weekends, we went on nature walks and explored the nearby mountain area. Later on, I decided to study biology and learn more about the natural habitat of wild flora and fauna. One of the most unforgettable experiences during my studies was an excursion to Kenya. We climbed up Mount Kenya up to 4500 m and explored the different vegetation zones with severe changes in temperature from day to night. This made me really curious about the underlying molecular mechanisms how plants can survive these temperature changes. From that point on I was interested in the molecular basis of life.

## When did you decide to become a scientist?

During my studies in biology with the major in plant biology and genetics, I decided to attend a two months internship in a field that was completely new to me so to open my mind to a different area of specialization. I was introduced to the – for me – new world of virology, and at that times, in the early 90s, still mainly unexplored and fascinating field in virology, human and primate lentiviruses. This was an eye-opening experience and changed my career path to molecular medicine. From that point on, it was clear I would stay in science and continue working on understanding the relationship of a virus and its host.

## When and where did you start your own lab?

My group is situated at the Paul-Ehrlich-Institute, the Federal Institute for Vaccines and Biomedicines, in Germany. I returned to my roots, the same place I had performed my internship as a young student, and now years later, in December 2010, established my own research group completing the circle.

## What areas or topics does your lab currently focus on?

My research continues to focus on HIV-1, in particular the intricate relationship between the virus and the cellular defense of the host cell. Innate immunity is the first line of defense against foreign pathogen invasion. Viruses must evade these host protective mechanisms to establish productive infections. Our current efforts are directed towards gaining a better understanding of the mechanism how a cell senses a virus and restricts its replication. Targeting these host-pathogen interactions is an attractive strategy for the development of novel immune-mediated antiviral therapies. We seek to elucidate the host factors usurped by viruses as well as innate immune factors sensing or counteracting viral replication through a systems approach. We use genome-wide RNAi screening technologies to identify cellular factors affecting virus replication and proteomics approaches that allow an unbiased construction of a pathogen-host interactome. In particular, we expanded our interest – besides HIV-1 – to Reverse-Transcribing viruses, such as HBV, and RNA viruses, such as Influenza and Ebola virus.

## Tell us about the most important stages of your professional career

After finishing my PhD in 2002, I joined the lab of Prof. Ned Landau at the Salk Institute in San Diego as a postdoctoral fellow to learn more about HIV-1-host-interactions. At that time, the first cellular factor that restricted HIV-1 was discovered, APOBEC3G. We studied how this factor influences the life cycle of HIV-1 and is counteracted by one of the HIV-1 accessory proteins. I enjoyed working in the US in one of the most prestigious academic labs, but I felt, before settling for an academic career, I should explore an industry setting and joined the Genomics Institute of the Novartis Research Foundation (GNF). There, I expanded my research on host-viral interactions on a global level through the application of systems-based approaches. GNF serves as a bridge between basic science & preclinical drug discovery and I gained a lot of experience in understanding the drug discovery process. I was excited to be able to apply state-of-the-art technologies to my research, and I realized that I really enjoy working on basic aspects and the molecular mechanisms. Later on, I decided to join my mentor, Prof. Sumit Chanda, to an academic setting, the SBP Medical Discovery Institute in San Diego, first as an Staff Scientist and later as Research Assistant Professor.

**Figure:**
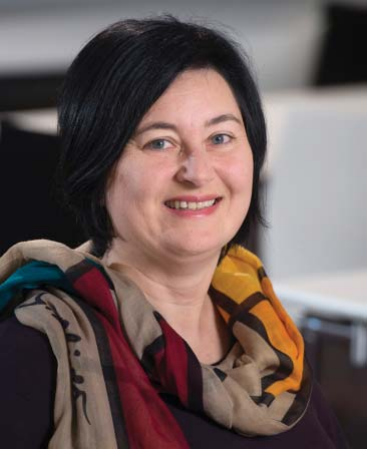

**About Renate König**. Dr. König is Head of Host-Pathogen Interactions unit at Federal Institute for Vaccines and Biomedicines, Paul Ehrlich Institute, Germany, and Adjunct Assistant Professor at Sanford Burnham Prebys Medical Discovery Institute, La Jolla, US. She received her PhD from Johann-Wolfgang-Goethe University of Frankfurt and Postdoctoral experience at the Salk Institute (Ned Landau lab), the Genomics Institute of Novartis and Sanford Burnham Prebys Medical Discovery Institute in San Diego (both in Sumit Chanda's lab). In her work, which focuses on viral infections and innate immune responses, Dr. König has employed high-throughput screens to identify virulence and host factors of HIV and influenza viruses. Her study of HIV-1 genetic factors for early-stage replication was selected as Faculty of 1000's Must Read in Medicine and Biology.

## What was your most significant scientific accomplishment?

Genomics research in collaboration with Sumit Chanda and John Young, has provided us with crucial knowledge of the cellular pathways and factors HIV-1 or Influenza virus exploit to replicate.^1,2^ Each of these represents an ‘Achilles heel’ of the virus and vastly increases the number of potential targets for new antiviral drugs. The HIV-1 study^1^ was one of the first papers to integrate genomics siRNA technologies with network analysis to map HIV's genetic flight plan during infection. The publication has been identified by *Science Watch®* in January 2011 as the most-cited paper in the category of Molecular Biology & Genetics. It was recognized as one of the key papers published in 2008 (Nature Medicine, Notable Advances, Dec. 2008) and got accepted into the Faculty of 1000 (Medicine and Biology) as a “Must Read”. Recent international collaborative efforts with Adolfo Garcia-Sastre, Silke Stertz and Sumit Chanda have resulted in successfully integrating results from various systems-level approaches in a Meta-Analysis to identify high confidence human proteins essential for Influenza replication.^3^ Further understanding of the roles for these proteins in virus infection will provide new insight into the host–pathogen interactions that orchestrate the viral replication cycle and new opportunities for the development of host-factor-directed antiviral therapies.

## What makes a good mentor?

Throughout my career, I have been privileged to work with excellent inspiring mentors. They believed in me and pushed me to go beyond what I thought I could accomplish and I gained confidence in my own abilities. I think, a great mentor can see your talent and can get the best out of you. He or she will connect you thoughtfully with the right people at the right time and support you in your first career steps.

## What advise would you have to junior people entering the field?

Pursue what you love doing. Don't shy away to follow an unconventional career path and “out of the box” thinking if this suits what is really important to you. The people you will meet along the way, the mentors and collaborators, the colleagues and friends, will always be an extremely important network for your career and for your personal life.

## What do you do for fun?

I still enjoy hiking and exploring the wildlife. During my postdoctoral times, I was part of the mountain rescue team in San Diego, a tremendous experience and training I still benefit from.

## References

[1] Konig R, Zhou Y, Elleder D, Diamond TL, Bonamy GM, Irelan JT, Chiang CY, Tu BP, De Jesus PD, Lilley CE, Seidel S, Opaluch AM, Caldwell JS, Weitzman MD, Kuhen KL, Bandyopadhyay S, Ideker T, Orth AP, Miraglia LJ, Bushman FD, Young JA, Chanda SK Global analysis of host-pathogen interactions that regulate early-stage HIV-1 replication. Cell. 2008;135:49-60. doi:10.1016/j.cell.2008.07.032. PMID:18854154 18854154 PMC2628946

[2] Konig R*, Stertz S*, Zhou Y, Inoue A, Hoffmann HH, Bhattacharyya S, Alamares JG, Tscherne DM, Ortigoza MB, Liang Y, Gao Q, Andrews SE, Bandyopadhyay S, De Jesus P, Tu BP, Pache L, Shih C, Orth A, Bonamy G, Miraglia L, Ideker T, Garcia-Sastre A, Young JA, Palese P, Shaw ML, Chanda SK Human host factors required for influenza virus replication. Nature. 2010;463:813-817. *contributed equally. doi:10.1038/nature08699. PMID:20027183 20027183 PMC2862546

[3] Tripathi S, Pohl MO, Zhou Y, Rodriguez-Frandsen A, Wang G, Stein DA, Moulton HM, DeJesus P, Che J, Mulder LC, Yanguez E, Andenmatten D, Pache L, Manicassamy B, Albrecht RA, Gonzalez MG, Nguyen Q, Brass A, Elledge S, White M, Shapira S, Hacohen N, Karlas A, Meyer TF, Shales M, Gatorano A, Johnson JR, Jang G, Johnson T, Verschueren E, Sanders D, Krogan N, Shaw M, Konig R#, Stertz S#, Garcia-Sastre A#, Chanda SK# Meta- and Orthogonal Integration of Influenza “OMICs” Data Defines a Role for UBR4 in Virus Budding. Cell Host Microbe. 2015;18:723-735. #co-corresponding authors. doi:10.1016/j.chom.2015.11.002. PMID:26651948 26651948 PMC4829074

